# Fat-tailed failure strength distributions and manufacturing defects in advanced composites

**DOI:** 10.1038/s41598-025-06693-4

**Published:** 2025-07-17

**Authors:** Rani Elhajjar

**Affiliations:** College of Engineering and Applied Science, University of Wisconsin-Milwaukee, 3200 N Cramer St, Milwaukee, WI 53211 USA

**Keywords:** Fiber waviness, Porosity, Defects, Multimodal distributions, Fat-tailed risk, Engineering, Materials science

## Abstract

This study investigates how manufacturing defects transform the statistical distribution of failure in carbon fiber-reinforced polymer composites under tension and compression loading. The analysis of tension and compression specimens reveals that defect-free composites exhibit relatively narrow, unimodal strength distributions. In contrast, specimens with porosity or fiber waviness develop more complex multimodal probability densities with fat-tailed distributions and substantially higher variability. Applying Jensen’s inequality demonstrates that this increased variability can be assessed to identify higher risk profiles. These findings indicate that defects in composites don’t simply reduce mean strength values but alter the statistical nature of composite failure, transforming thin-tailed, unimodal, well-behaved distributions into multimodal, fat-tailed ones. Such transformation necessitates more sophisticated probabilistic approaches for reliable design and strength prediction in safety-critical applications where understanding tail risk becomes crucial to proper risk management.

## Introduction

**Fig. 1:**
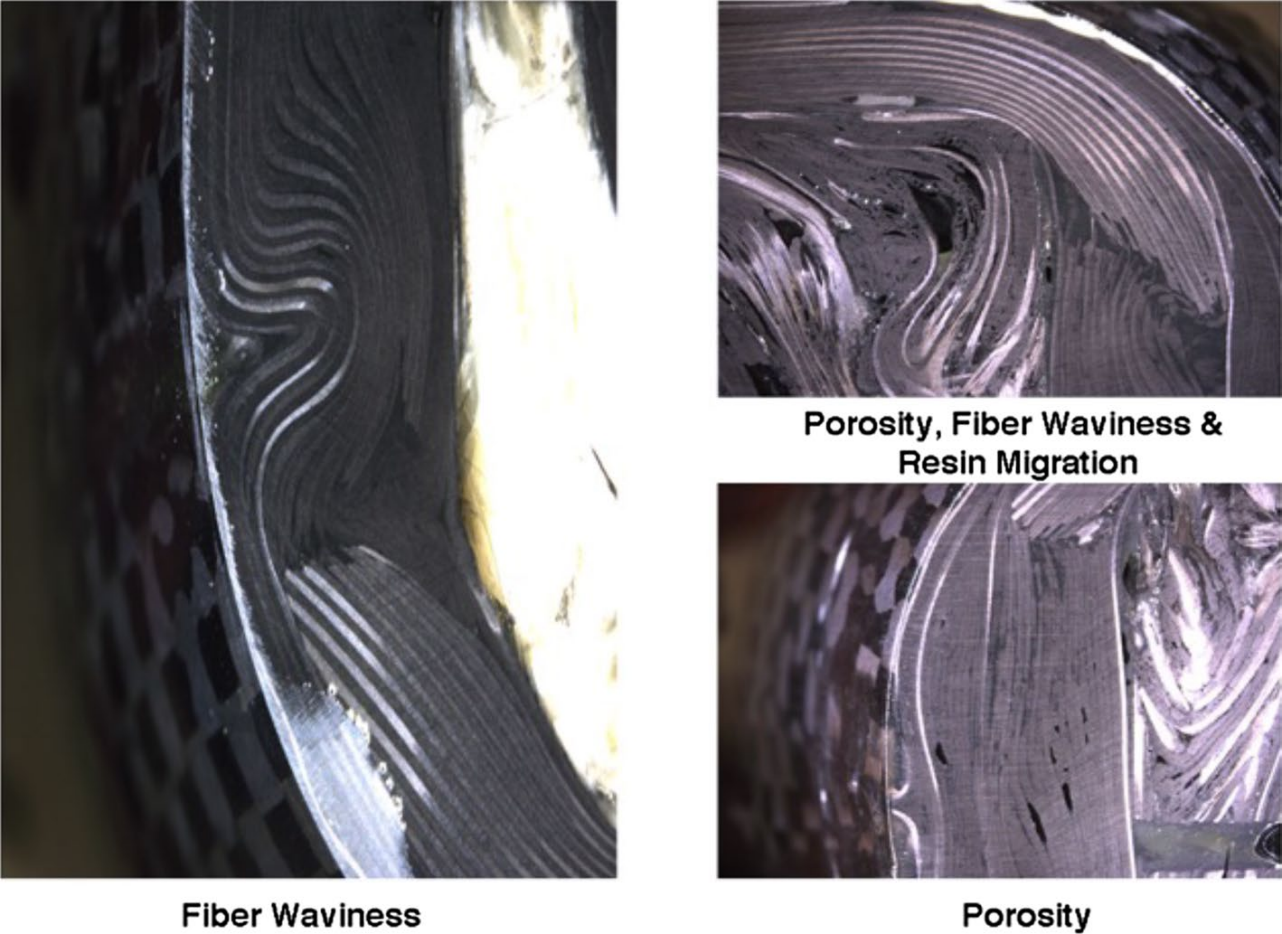
Microstructural defects in a carbon fiber reinforced composite bicycle frame. Optical micrographs of manufacturing defects that significantly alter strength distributions. Left: In-plane fiber waviness showing sinusoidal fiber misalignment. Top right: Combined defects showing the interaction between porosity (dark voids), fiber waviness, and resin-rich regions resulting from resin migration. Bottom right: Porosity defects appearing as dark voids between fiber tows^4^.

Manufacturing defects or imperfections are known to alter the failure mechanisms in fiber-reinforced composites and are practically unavoidable in industrial settings^1–4^. The prevalence of these imperfections increases substantially when designs include ply drops associated with thickness changes or in environments when using non-autoclave processing methods, building large structures with curved geometries, or using thick laminates (e.g., Fig. 1). Composite specimens can show considerable variability with coefficients of variation ranging from 3 to 20% in flat composites fabricated under ideal conditions compared to 2 to 4% for aircraft-grade sheet al.uminum^5^. This variability can be attributed to the composite materials being highly process-dependent due to their design and the need for precise control during multiple stages of fiber and resin production, tooling and preparation, layup, resin infusion or preimpregnation, curing, and post-processing.

Of the many possible defects or imperfections, the literature has noted the significant property reductions associated with porosity and fiber waviness. Porosity is widely recognized as a critical defect in advanced composites, with numerous studies documenting its detrimental effects on mechanical properties. Selected representative works^6–15^demonstrate how porosity undermines tensile and compressive strength by introducing localized stress concentrations, weakening the fiber-matrix interface, and changing the constitutive properties. The volume fraction and pore morphology (size, shape, distribution) dictate the severity of these effects, with larger pores or higher void content creates localized stress concentrations. This occurs if pores are irregularly shaped or clustered, which may initiate microcracks under tensile loads. Under compressive loads, porosity compromises the strength by destabilizing the matrix’s ability to support fibers against buckling and shear-driven failure. Voids reduce the matrix’s shear modulus and strength, weakening its capacity to stabilize fibers and resist micro buckling.

In contrast, as seen in selected representative works^16–25^fiber waviness diminishes the tensile and compression strength by disrupting load transfer efficiency and inducing localized stress. In tension, the misaligned fibers that deviate from the optimal axial orientation force the weaker matrix to bear excessive loads, leading to accelerated matrix cracking and interfacial debonding. The severity of the waviness defect can be characterized by the wavelength-to-amplitude relationship, angle, or depth to the thickness of the laminate. In essence, severe and deep undulations create concentrated bending stresses at wave peaks and troughs, exacerbating shear-lag effects and matrix failure. These sharp bends also subject fibers to transverse bending stresses, which they are poorly equipped to resist, leading to premature fiber breakage. Higher misalignment angles or those also with higher depth or amplitudes further shift stress to the matrix, promoting early debonding. Fiber waviness destabilizes the composite in compression by promoting micro-buckling and kink-band formation^26^. Fiber undulations create geometric “hotspots” where sharp fiber curvature reduces the critical buckling stress, triggering localized micro-buckling. The angle of waviness and, in corollary, the depth of the waviness is particularly detrimental here, as per kink-band theory: even minor misalignment angles (2–5°) lower the critical stress required for shear-driven kinking, where fibers buckle and collapse into narrow failure bands. The matrix, strained by amplified shear stresses near wavy fibers, undergoes significant stresses and failure, further destabilizing the structure. Additionally, fiber waviness reduces the composite’s local effective stiffness, allowing more significant deformations that accelerate buckling. Compression strength is thus far more sensitive to waviness as failure hinges on alignment-sensitive mechanisms like kink band formation.

Probability density functions provide a quantitative framework to describe how structural imperfections transform strength distributions beyond simple mean reductions. Previous studies have utilized Weibull^27,28^ and stochastic simulations^16,29–33^ as statistical tools to capture variability in the mechanical properties of composite materials. Linking specific defect parameters to performance degradation often assumes unimodal distributions that may inadequately represent the true complexity of failure mechanisms. Furthermore, these approaches fail to account for the mathematical consequences of nonlinear relationships between defect parameters and strength properties. Jensen’s inequality, a fundamental principle in probability theory, demonstrates that for concave functions, the expected value of the function is less than or equal to the function of the expected value $E[f(X)\le f(E[X])$ . This inequality has essential implications for composites with manufacturing variability, as it explains why even minor defect variations can produce disproportionate strength reductions when the strength-defect relationship is concave. Thus, the tilt towards fat tail distributions can increase risk and reduce reliability in concave functions^34,35^.

Through mechanical testing of tension and compression specimens with controlled defect introduction, we demonstrate that porosity and fiber waviness manufacturing defects alter the probabilistic landscape of composite failure. Using a mixed-mixture model, we show that fiber waviness and porosity generate multimodal strength distributions with distinct failure signatures that conventional single-parameter statistical approaches cannot adequately capture. This multimodal distribution is particularly evident in compression loading, where fiber waviness creates early failure modes that appear as significant left-tailed outliers in the strength distribution and increase Jensen’s gap. These findings reveal the need for a critical look at approaches based on mean values of correction factors based on assumed Gaussian or Weibull distributions, as they may underestimate the probability of premature failure in defect-containing structures. By accurately characterizing the transformation from unimodal to multimodal distributions, engineers can develop more robust designs that properly account for the risk profile of composite structures, particularly those dominated by compression loading.

## Methods

A total of one hundred and eighty specimens were included in this study, with ninety being tested in tension and ninety specimens being tested in compression. In each tension or compression group, thirty specimens were in the baseline or control group, thirty with porosity induced during manufacturing (porosity group), and thirty specimens with out-of-plane fiber waviness induced in the manufacturing process (fiber waviness group). The specimens were fabricated under controlled laboratory conditions to systematically investigate the relationship between the design parameters and mechanical performance. While production environments may exhibit a different variability in defect morphologies (depending on a wide range of manufacturing factors), the approach used intentionally isolated specific structural variables (porosity fraction percentage, and depth/thickness ratios of fiber waviness) to establish strength-structure relationships. This controlled experimental design is done to study the relationship between normalized strength and defect parameters and whether a disproportionate strength reduction exists when structural variability increases. The relationships and the underlying mechanics would apply to diverse defect morphologies, though the specific parameters may vary with manufacturing procyesses. The composite is fabricated from prepreg form and comprises a Toray T700GC-12 K-31E carbon fiber with the #2510 epoxy system prepreg (Toray Composites, Tokyo, Japan) with a reported fiber volume fraction of 54.4%^36^. The prepreg form is selected because it reduces the variability in the process, and the resin amount is precisely controlled compared to other methods like resin infusion or wet layup. The prepreg plies were collated into the following 16-ply layup for waviness [0/45/90/−45/0/45/−45/0]_s_ and 10-ply layup [0/45/90/−45/0]_s_ for the porosity specimens. The specimens were cured in a compression hot press (GE30H-15-BCX, Wabash, IN, USA) with a single hold per the manufacturer recommendations where the heating and cooling rates were 1 degree C/min and 2.5 degrees C per minute, respectively, and a hold of two hours at 132 degrees C and the maximum pressure applied for the specimens is 100 kPa during the hold. This pressure was reduced for the porosity specimens to different levels in a trial-and-error approach to encourage more void formation. For each layup configuration, fifteen baseline specimens were prepared with the identical stacking sequence but without the intentionally introduced defects. All strength values reported in the study were normalized to their respective group’s baseline mean value, ensuring that differences in laminate sequence did not affect the conclusions regarding defect impact. This normalization approach isolates the effect of the defects from the inherent differences between layup configurations. We found that different defect types require different optimal layups for proper manifestation and characterization, with fiber waviness needing greater thickness (16 plies) for meaningful depth-to-thickness ratios while porosity is better controlled and analyzed in thinner laminates (10 plies).

**Fig. 2:**
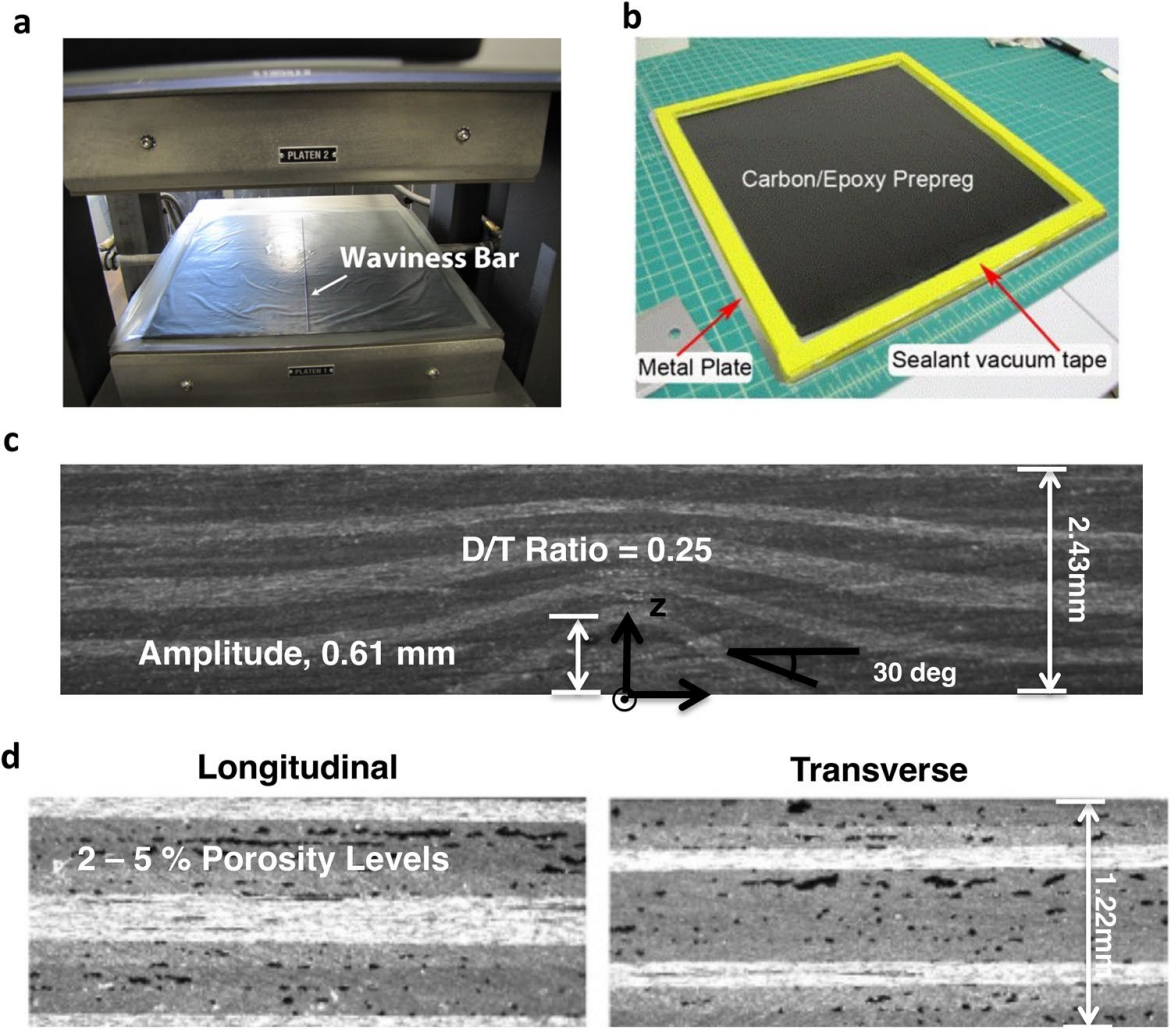
Manufacturing methods and characterization of composite specimens with controlled defects. (**a**) Hot press setup showing the placement of a waviness bar on the uncured composite laminate to induce controlled fiber waviness; the bar is removed before final curing. (**b**) Preparation of a porosity specimen with carbon/epoxy prepreg sealed between metal plates using vacuum sealant tape to trap gases during curing. (**c**) Cross-sectional image of a composite with quantified surface waviness parameters with a depth-to-thickness ratio of 0.25. The coordinate system and 30° angle orientation are indicated. (**d**) Comparative microscopy of longitudinal and transverse sections showing different porosity ranges.

The fiber waviness specimens were manufactured using small diameter rods to disturb the plies separated from the composite using a Teflon sheet, as described in the procedure described in Elhajjar and Peterson^18^ as shown in Fig. 2a. The experimental design focused on isolating the effect of a single out-of-plane fiber waviness defect with depth-to-thickness ratios ranging from approximately 0.03 to 0.3. This controlled approach allowed for direct comparison between the baseline, porosity, and waviness groups without the confounding effects of multiple interacting waviness defects. Porosity in fiber-reinforced polymer composites refers to the presence of unintended void spaces or gas-filled cavities within the laminate. These voids typically form during the manufacturing process when air or other gases become trapped between fiber layers, within fiber bundles, or in the matrix material itself.

The porosity specimens were manufactured by sealing the uncured panel between two plates to entrap and increase the internal pressure inside the panel using the procedure previously outlined by Peng and Elhajjar^8^ (Fig. 2b). Cross-sectional grayscale analysis for porosity assessment is made by establishing appropriate grayscale thresholds on polished specimens to estimate void content even when waviness is present. In the study, porosity levels ranged from below 2% in baseline specimens to approximately 10% in the porosity group, quantified through cross-sectional grayscale analysis where darker regions in optical micrographs correspond to void spaces. Porosity is quantitatively expressed as a percentage (volume fraction), representing the ratio of voids to the total composite pixel count. Examining both longitudinal and transverse sections on the panels enhances characterization by showing how porosity manifests differently across orientations while simultaneously allowing independent measurement of waviness profiles. Figure 2c and d show representative cross-sections for the cut and polished edges of the specimens fabricated. The images display the characteristic appearance of fiber waviness and porosity defects deliberately introduced during manufacturing. Fiber waviness is also characterized by using optical images and measuring the depth to thickness ratio using pixel counts. The specimens in the baseline group had porosity levels below 2% and no detectable fiber waviness, whereas those in the porosity group would have up to 10% porosity levels. The specimens were then machined and tested according to ASTM D3039^37^ for the tension specimens and ASTM D6641^38^ for the compression specimens. After the testing, the results were normalized to the baseline for comparative purposes.

For the waviness specimens, the depth-to-thickness ratio was selected as the waviness characterization metric based on both its physical significance in relation to failure mechanisms and its practical advantages for characterization. This dimensionless parameter quantifies out-of-plane fiber waviness by measuring the maximum perpendicular deviation of the fiber from its intended path (depth) relative to the total laminate thickness. The depth-to-thickness ratio connects directly to mechanical principles through established equations for buckling and kink-band formation. According to the modified Argon equation in Budiansky and Fleck^39^the critical compressive strength $(\sigma_{cr})$ for a composite with elastic-perfectly plastic behavior, with fiber misalignment can be approximated by:


1


where *G* is the shear modulus, and $\phi_0$, is the maximum initial fiber misalignment angle, and, $\gamma_y$ is the yield shear strain. From a practical standpoint, the wavelength measurements in manufactured components often show high variability, making reliable angle calculations challenging. The depth-to-thickness ratio provides a more robust metric that can be consistently measured from cross-sectional images and links not only a measure of the distortion through the maximum amplitude of the waviness but also the relative distortion within the laminate through the thickness measurements.

## Results and discussion

**Fig. 3:**
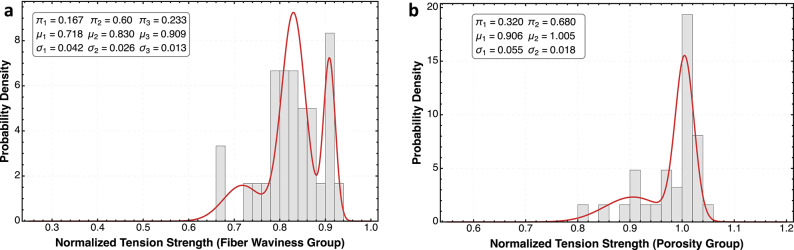
Probability density distributions of normalized tension strength for composite laminates with different defect types (**a**, **b**). The plot in (**a**) illustrates the statistical distributions of tension strength normalized to the baseline for fiber waviness, which results in the most significant strength reduction, displaying a complex multimodal distribution with primary peaks at approximately 0.85 and 0.9 and a minor mode near 0.7. In (**b**), the porosity-containing specimens demonstrate a notably higher peak with a narrower distribution, suggesting reduced variability but with a strong bimodal character, including a secondary peak around 0.9.

**Fig. 4:**
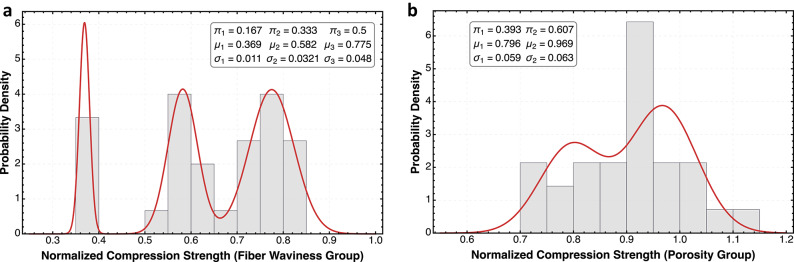
Probability density distributions of normalized compression strength for composite laminates with different defect types. The plot in (**a**) displays the statistical distributions of compression strength normalized to the baseline for fiber waviness, which shows the most dramatic strength reduction with a complex multimodal pattern featuring a dominant peak at approximately 0.4 and additional modes at 0.6 and 0.75. The plot in (**b**) shows the porosity-containing specimens demonstrating a multimodal distribution with peaks at approximately 0.80, and 0.97, indicating variable performance effects.

The curves in Figs. 3 and 4 show probability density functions fitted to experimental strength data for the tested composite specimens. After conducting the standardized unnotched compression and tension tests on multiple specimens from each condition, the raw strength data was organized into histogram bins to visualize the empirical distribution. The data was modeled using a Gaussian mixture model^40^ with the probability density function given by:


2


where $\pi_k, \mu_k,$ and $\sigma_k^2$ are the weight, mean, and variance of the k-th component, respectively of the distribution, and each Gaussian PDF given by:


3


The statistical analysis of both the tension and compression strength distributions reveals interesting insights into defect-related failure mechanisms. The baseline material exhibits nearly Gaussian distributions centered around normalized strengths of 1.0 for both loading conditions and across both laminates, indicating consistent defect free well-manufactured composites. However, this unimodal behavior dramatically changes to multimodal behavior when defects are introduced, with compression properties changing the probability distribution more significantly than tension (Figs. 3 and 4).

**Fig. 5:**
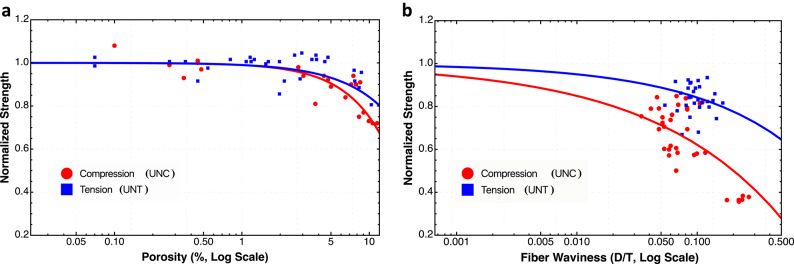
Normalized strength as a function of porosity (a) and wrinkle depth/thickness ratio (b) under compression (red) and tension (blue) loading. Both relationships demonstrate concavity, which, according to Jensen’s inequality (, for concave functions), explains why defect variability disproportionately reduces strength compared to uniform defects with the same average values. The more pronounced concavity in compression demonstrates mathematically why compression properties are more sensitive to manufacturing than tension properties. Note the logarithmic scale on both x-axes.

The strength degradation observed in the defect-containing specimens exhibits helps explain why manufacturing variability is particularly detrimental to composite reliability. As shown in Fig. 5, the relationship between normalized strength and defect parameters (porosity and waviness depth/thickness ratio) follows a concave function, particularly pronounced for compression loading illustrating how small changes in defect profile at the upper edges results in even larger drops in composite strength. This concavity is directly connected to Jensen’s inequality, which states that for a concave function $f(x)$ , the expected value of the function is less than or equal to the function of the expected value:


4


For the composite strength data, if we define *X* as the defect parameter (either porosity or the depth to the thickness of the waviness defect) and $f(X)$ as the resulting strength, this inequality can be used to understand how the manufacturing variability produces disproportionate strength reductions under variability. Consider two composite regions with defect parameters $x_{1}$ and $x_{2}$, where their average is:


5


Since the strength-defect relationship is concave (as shown in Fig. 5), then:.


6


This observation reveals a critical insight that the composite with uniform defect distribution at parameter value $\overline{x}$ will be stronger than a composite with the same average defect parameter but with local variations between $x_{1}$ and $x_{2}$ . The difference between these two quantities, often called the *Jensen gap*, increases with both the function’s concavity and the defect distribution’s variance. The practical consequence is that controlling defect variability is as important as controlling the mean defect parameters. The data shows that the concavity is more pronounced for compression strength than for tension strength (Fig. 5), which mathematically explains why compression properties are more sensitive to manufacturing defects and is seen in the multiple modes observed. For waviness specifically, the concave relationship demonstrates that a small number of severe waviness defects will degrade the overall composite strength more than would be predicted by the average waviness parameter, creating the fat-tailed strength distributions observed in Figs. 3 and 4. The Jensen gap can thus be quantified as:


7


Jensen’s gap highlights that estimates of strength based on average defect parameters may overestimate the actual strength of variable-quality composites, particularly under compression loading and fiber waviness defects where the strength-defect relationship exhibits greater concavity. The results show fiber waviness creates the most drastic reductions in both loading modes. In tension (Fig. 3a), waviness produces a complex multimodal distribution with peaks between 0.7 and 0.9, suggesting several competing failure mechanisms. In compression (Fig. 4a), the effect is even more severe, with three distinct modes, including a failure peak at approximately 0.4, representing a 60% reduction in strength compared to the baseline. This trimodal distribution indicates different failure pathways activated by fiber misalignment, with the compression failure mechanism likely involving micro-buckling of misaligned fibers at substantially lower loads than in pristine material. Porosity presents an interesting contrast between tension and compression, both having a bimodal distribution with more strength reduction in compression. The leftward shift of the distribution indicates that porosity more severely impacts compression than tension properties, though not as dramatically as fiber waviness does. While waviness introduction does create localized resin-rich regions at the distortion point, we can successfully isolate waviness as the primary defect variable, keeping porosity levels below the threshold of 2% in those specimens. This isolation of variables supports the conclusion that the multimodal strength distributions observed in the waviness group can be attributed primarily to fiber misalignment rather than to secondary porosity effects from porosity interactions, even though we cannot rule out that some porosity may be present. The direct comparison across all conditions reveals critical differences in statistical behavior, with compression testing demonstrating greater sensitivity to manufacturing defects. Fiber waviness introduces the most profound changes in the probability density function, particularly in compression, where the earliest normalized strength at 0.4 results in a severe fat-tailed distribution.

Fiber waviness creates fat-tailed distributions under compression loading due to the instability mechanisms involved in compressive failure of composites. Unlike tension loading where fibers resist primarily through their material strength, compression loading triggers stability-dependent failure modes that are sensitive to misalignment. When fibers are wavy, compression forces create moments that amplify rather than straighten the initial misalignment, initiating a self-reinforcing failure cascade. As shown in Fig. 5b, the relationship between normalized strength and waviness depth/thickness ratio is strongly concave in compression, creating distinct threshold regions where small increases in waviness severity cause disproportionately large strength reductions. This concavity, combined with the inevitable variability in manufacturing-induced waviness, mathematically generates fat-tailed distributions through Jensen’s inequality (Eq. 7). The trimodal distribution observed in Fig. 4a reflects these different failure mechanisms: specimens with severe waviness trigger early kink-band formation at approximately 0.4 normalized strength, creating the leftmost mode, while specimens with milder waviness exhibit different failure pathways represented by the modes at 0.6 and 0.75.

The defects reshape composite strength distributions by activating distinct failure mechanisms that manifest as separate statistical modes. Porosity transforms the baseline unimodal distribution into a bimodal pattern in both tension and compression (Figs. 3b and 4b), with the bimodality increasing as void content approaches 10%. This results from varied void morphologies creating different stress concentration effects, void clustering forming localized weakened regions, and interactions between voids and the fiber architecture. Fiber waviness produces more dramatic distribution transformations, particularly under compression where a pronounced trimodal distribution emerges with modes at approximately 0.4, 0.6, and 0.75 normalized strength (Fig. 4a). This trimodality reflects how the waviness severity creates distinct failure thresholds where small parameter increases cause disproportionate strength reductions. As defect severity increases, the distribution exhibits greater variability with the leftmost mode growing from a minor component to a dominant feature, while the overall distribution develops a fat left tail. The concave relationships shown in Fig. 5 mathematically explain this transformation through Jensen’s inequality - as defect variability increases, the “Jensen gap” widens, creating greater disparity between the expected strength based on average defect parameters and the actual strength of variable-quality composites. Conventional statistical approaches based on mean values may significantly underestimate failure probabilities in defect-containing structures, particularly under compression loading where the concavity is most pronounced.

This finding aligns with fat-tailed risk distributions, where rare events (specimens with particularly severe waviness or high porosity) disproportionately impact structural reliability. While a study with tightly controlled, identical waviness parameters might yield narrower distributions related to different failure modes, such uniformity would misrepresent the inherent variability encountered in industrial manufacturing processes. A central point to highlight is that the included waviness profiles are within the range of possible manufacturing conditions. The practical challenge remains that composite production cannot perfectly control fiber alignment throughout complex structures, nor can inspection methods feasibly identify every waviness defect and its severity despite constant improvements. Therefore, the fat-tailed distributions observed in testing provide a realistic representation of composite structures’ risk profile, highlighting why conventional statistical approaches based on Gaussian assumptions and standard deviations may significantly underestimate failure probabilities. Given that manufacturing processes cannot guarantee that fiber waviness defects can be eliminated, the statistical nature of composite failure with fiber waviness should be considered. Considering the possibility of other defects in materials, manufacturing and service, it is expected that the concavity in the response will be further pronounced creating additional challenges in for design and reliability of composite structures.

Advanced manufacturing techniques with precise fiber placement control, combined with inline quality, can move us up the strength-defect curve but cannot eliminate the concave nature of this relationship. Any slips in quality control or inspections could yield additional variability and exposure to increased risk. Achieving advanced composite manufacturing perfection remains aspirational, particularly for complex geometries, thick parts, and large-scale structures where complete elimination of waviness may be physically and economically unattainable. Until such manufacturing breakthroughs are realized, design approaches must continue to account for the risk profile demonstrated in this study. When multiple defect types interact, the multimodal nature of strength distributions necessitates the need for additional redundancies and probabilistic approaches that properly account for tail behavior rather than central tendencies.

When analyzing how defects in composites interact, it is also essential to characterize not merely their individual statistical properties but the functional relationship between them. The interaction mechanism may be additive (where combined effects are summed), multiplicative (where effects amplify each other), or involve more complex nonlinear dynamics that lead to negative feedback loops. For instance, when examining the interaction between manufacturing defects like porosity and fiber waviness, the relationship of each one follows a concave function. A combined effect may result in disproportionate strength reductions compared to what would be predicted by examining each defect in isolation. Jensen’s inequality (Eq. 4) may also provide a critical framework here, demonstrating that the expected value of a concave function is less than or equal to the function of the expected value. This gap between these values widens as the concavity increases and as the distributions become more variable or exhibit fatter tails. Special attention must be paid to their potential for amplification effects where one defect triggers others through mechanisms similar to those observed in cascade failures, potentially transforming thin-tailed risks into more severe fat-tailed ones.

In loading conditions other than the uniaxial loading considered in this study, manufacturing defects will likely affect composite strength distributions through a more complex mechanism than in pure tension or compression. For example, in bending, both tensile and compressive stress are present across the thickness. The location of defects relative to the neutral axis would significantly influence failure initiation and progression, with defects in the compression zone having disproportionately greater impact than those in the tension zone. This spatial dependency would likely introduce additional modes in the strength distribution beyond what we observed in pure tension or compression separately. The concavity of the strength-defect relationship may potentially be amplified in bending, further widening the Jensen gap (Eq. 7) and increasing the fat-tailed characteristics.

These cases of different loading modes or coupled defects, highlight the limitations of traditional approaches for using mean-based reductions from defect-containing composites, especially under compression loading. The multimodal distributions observed suggest different physical mechanisms are activated depending on defect type, severity, and loading condition, necessitating more sophisticated probabilistic approaches that can account for these complex, non-Gaussian behaviors. For critical non-redundant structural components where compression loading is significant, particular attention must be paid to fiber waviness, as its propensity to generate early failures creates a reliability profile different from what conventional statistical models would predict based on the baseline defect-free model.

## Conclusions

This article demonstrates that manufacturing defects in fiber-reinforced polymer composites transform strength distributions from unimodal Gaussian patterns to complex multimodal distributions. Most critically, fiber waviness in compression loading generates significant fat-tailed distributions where failure probabilities are substantially higher than traditional models predict.

While previous work has addressed the detrimental impact of manufacturing defects on composite strength, the research provides several novel insights that extend the conventional understanding that defects merely lower mean strength and increase variability. This study shows that rather than simply shifting or widening existing distributions, we demonstrate that the wrinkle and porosity manufacturing defects show multimodal probability densities with fat tails. This represents a qualitative change in distribution type from unimodal distributions and not merely a quantitative reduction in strength parameters. The results show that different defect types (porosity versus waviness) and loading conditions (tension versus compression) create distinctive multimodal signatures in the probability density functions. These signatures reflect different failure mechanisms rather than simple shifts in a single distribution, with compression showing particularly pronounced multimodality with fiber waviness. The fat-tailed distributions we identify have profound implications for reliability engineering that go beyond traditional safety factors. While conventional approaches might consider a 3-sigma or 6-sigma design margin adequate, the findings demonstrate that such approaches may underestimate the probability of failure when distributions have fat tails, particularly with fiber waviness under compression. The application of multimodal Gaussian mixture models combined with Jensen’s inequality show introduces a practical quantitative methodology to estimate the “Jensen gap” (Eq. 7) as a function of defect variability - a direct measure of how much conventional mean-based property estimates may overpredict actual strength in defect-containing composites. This approach also provides designers with a novel quantitative tool, allowing for more accurate risk assessment.

The controlled nature of specimen preparation represents a strength and limitation of this study. While the approach may not capture the full spectrum of defect morphologies encountered in varied production environments, it provides a crucial insight into the mechanics governing strength reduction in the presence of manufacturing defects in composites. The clear concave relationship between normalized strength and defect parameters aligns with Jensen’s inequality, suggesting that this mathematical framework can be used to understand how manufacturing variability affects mechanical performance. Future work could extend this analysis to more diverse production methods, potentially yielding refined parameters while still operating within the same mathematical framework established here.

In addition, the risk from these defects can be managed through more enhanced inspection techniques that could identify critical defects, though such detection remains challenging, particularly for fiber waviness. Simultaneously, design methodologies must evolve beyond traditional knockdown factors based on averages toward frameworks that explicitly account for fat-tailed distributions, possibly including approaches from extreme value theory, reliability-based optimization for multimodal failure mechanisms, and increased structural redundancy in compression-dominated components. Conventional safety factors derived from mean values are likely inadequate when distributions have heavy tails, as risk becomes dominated by variability, leading to possibilities of rare but severe defects. The multimodal nature of strength distributions in composites with manufacturing defects necessitates probabilistic approaches that properly account for tail behavior rather than central tendencies.

## Data Availability

Data sets generated during the current study are available from the corresponding author on reasonable request.
